# Cavity-Filling Mutations as a Strategy to Stabilize Prefusion Viral Fusion Proteins for Vaccine Design

**DOI:** 10.34133/csbj.0199

**Published:** 2026-08-19

**Authors:** Reetesh Kumar, Pandiyan Muthuramalingam, Savitri Tiwari, Jyoti Gupta, Rohan Gupta, Prashant Agrawal, Himanshu Yadav, Naveen Kumar, Janhvi Mishra Rawat, Karthikeyan Ravi, Hyunsuk Shin

**Affiliations:** ^1^ Department of Bioengineering and Biotechnology, School of Biosciences & Technology, Galgotias University, Greater Noida, Uttar Pradesh 203201, India.; ^2^ Department of Horticultural Science, Gyeongsang National University, Jinju, South Korea.; ^3^ Department of Life Sciences, School of Biosciences & Technology, Galgotias University, Gautam Buddha Nagar, Greater Noida 201310, India.; ^4^ Department of Biotechnology, GLA University, Mathura 281406, India.; ^5^ School of Forensic Sciences, Galgotias University, Greater Noida 201310, Uttar Pradesh, India.; ^6^ Department of Forensic Science, Sharda School of Allied Health Sciences, Sharda University, Greater Noida 201310, Uttar Pradesh, India.; ^7^ School of Basic & Applied Sciences, Galgotias University, Greater Noida 201310, Uttar Pradesh, India.; ^8^ Department of Biotechnology, Graphic Era Deemed to be University, Dehradun 248002, Uttrakhand, India.; ^9^ Centre for Herbal Pharmacology and Environmental Sustainability, Chettinad Hospital and Research Institute, Chettinad Academy of Research and Education, Kelambakkam 603103, Tamil Nadu, India.

## Abstract

Enveloped viruses use metastable fusion proteins to enter host cells through large-scale conformational rearrangements. The prefusion conformation represents a higher-energy metastable state that irreversibly refolds into a lower-energy postfusion conformation during membrane fusion. Although the prefusion state contains the key neutralizing epitopes, it represents an ideal target for vaccine development. However, because of the instability of the prefusion state, the development of a viable vaccine and the determination of the prefusion state are both major challenges. Building on advances in structure-based vaccine design, cavity-filling mutations have emerged as a promising strategy for stabilizing prefusion viral fusion proteins across viral systems. Cavity-filling mutations have been shown in several viral systems to improve protein stability, thermostability, antigen expression, and immunogenicity. However, these effects depend on the structural architecture, cavity geometry, and local conformational context of each protein. In addition, these modifications may reduce premature transitions to the postfusion conformation and may help preserve critical neutralizing epitopes, depending on the structural context, while maintaining a metastable prefusion state. Accordingly, this review summarizes the molecular basis of cavity-filling-mediated stabilization, highlights successful applications across diverse viral families, examines how structural stabilization influences immunogenicity, and discusses the potential of cavity-filling strategies for rational vaccine design.

## Graphical Abstract

**Figure:**
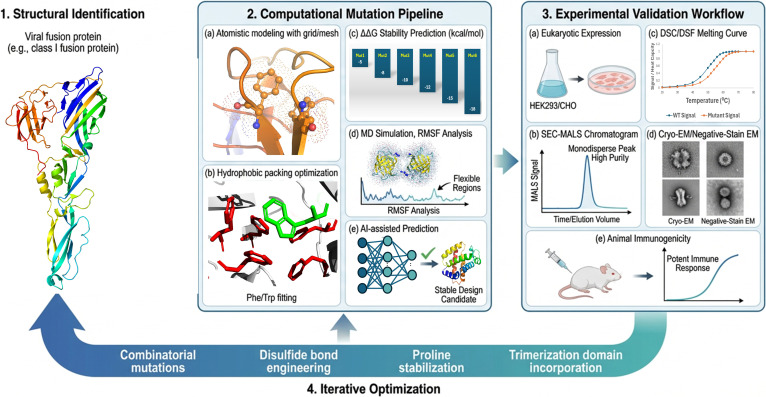

## Introduction

### The challenge of viral fusion proteins

Viral fusion proteins are among the most complex and dynamic molecular assemblies found in biological systems. They mediate membrane fusion, a critical process that enables viral genomes to enter host cells [1]. To accomplish this function, fusion proteins must satisfy 2 seemingly contradictory requirements: They must be sufficiently stable to withstand extracellular conditions during viral maturation and transmission while retaining the conformational flexibility necessary to undergo extensive structural rearrangements during membrane fusion [2]. To satisfy both requirements of structural integrity and activation readiness, viral fusion proteins have evolved a metastable prefusion state that balances structural integrity with activation readiness [1]. Structurally, most class I viral fusion proteins, such as hemagglutinin (HA) protein of influenza virus, F protein of respiratory syncytial virus (RSV), spike (S) protein of coronaviruses, and glycoprotein (GP) protein of filoviruses, are produced in a precursor form that undergoes proteolytic processing before becoming fusion competent [3,4]. The prefusion state represents a compact, high-energy metastable configuration that eventually converts into a lower-energy postfusion state during membrane fusion. Activation is triggered by virus-specific stimuli, including receptor binding, pH changes, or proteolytic processing, which initiate large-scale conformational rearrangements [5,6]. The structural and energetic basis of this transition is discussed in The prefusion-to-postfusion transition: Structural and energetic basis of conformational rearrangement section. The conversion from the prefusion to the postfusion conformation will release the conformational energy needed to facilitate membrane fusion and entry of the virus into host cells [2].

The intrinsic instability of viral fusion proteins poses a major challenge for vaccine development and antigen design [7]. Many potent neutralizing antibodies recognize epitopes that are uniquely displayed in the prefusion conformation [8–10]. Because this state is metastable, recombinant proteins frequently undergo spontaneous conversion to the postfusion form during expression, purification, or storage [2,11]. Consequently, postfusion antigens often elicit predominantly non-neutralizing or less potent antibody responses [12]. Similar challenges have been observed across multiple viral families, highlighting the importance of prefusion stabilization strategies for vaccine antigen design [13]. Therefore, maintaining the prefusion conformation throughout antigen production is essential for preserving protective epitopes and achieving optimal vaccine efficacy [14,15]. Understanding the structural determinants of prefusion instability has therefore become a central objective of structure-based vaccine design and forms the foundation for stabilization strategies discussed throughout this review.

### Historical context and evolution of stabilization strategies

Efforts to maintain viral fusion proteins in their prefusion conformations have been influenced by developments in technology and past experiences. One example is the formaldehyde-inactivated RSV vaccine of the 1960s, which had an unexpected outcome upon subsequent natural infection after vaccination; the individuals experienced worse respiratory illnesses than those who received the live virus [16]. This was attributed to the formation of misfolded F-protein during the inactivation procedure, illustrating the importance of preserving the native conformation of fusion proteins intact when designing vaccines [17,18]. For many years, the lack of detailed information on the structure of fusion proteins has limited the ability to systematically try to stabilize them. Cryo-electron microscopy (cryo-EM) and x-ray crystallography have greatly improved our understanding of the detailed 3-dimensional structure of fusion proteins at both the prefusion and postfusion conformations in several viral families [1,19–21]. Structural studies revealed the major changes in the conformation of the proteins necessary for membrane fusion and that often require the unfolding of helical bundles and extensive movement of large domains [7,22]. More importantly, structural studies identified specific locations within the protein that are responsible for destabilizing the prefusion state. Following this knowledge, Kumar *et al*. [7] developed new structure-based design techniques to stabilize fusion proteins in a prefusion state. Initially, structure-based stabilization strategies focused on introducing disulfide bridges to restrict the motion of key domains. Subsequently, 2 proline-based mutation strategies, 2P and HexaPro, were developed to stabilize hinge regions in coronavirus S proteins [23–25]. Similar proline-based approaches were also successfully applied to class I viral fusion proteins from other viruses, including RSV F, which improved both their stability and immunogenicity in the prefusion state [26]. However, while proline substitutions and disulfide linkages produced meaningful results for some fusion proteins, subsequent studies demonstrated that not all fusion proteins respond similarly to such methods. Therefore, the cavity-filling strategy represents an evolution from largely empirical approaches toward more rational, structure-guided methodologies to stabilizing proteins. Thus, researchers have begun to study new and more subtle approaches to stabilizing fusion proteins. The search for new and refined ways to stabilize fusion proteins has led researchers to explore other ideas that are based on protein thermodynamics and structural optimization. Recent advances in structure-guided computational stabilization strategies have further expanded opportunities for rational vaccine design [4,7,25]. Among these strategies, cavity-filling-mediated prefusion stabilization has emerged as one of the most promising approaches.

### Cavity-mediated instability and the rationale for cavity-filling

Most viral fusion proteins have a prefusion structure with internal cavities. Internal cavities are small, empty spaces inside the hydrophobic core of the prefusion structure, which can contribute to prefusion metastability [2,3]. The smaller the size of the internal cavities within the prefusion structure, the lower the packing efficiency of the structure. The less efficiently packed the structure is, the more local strain will be developed in the structure as it is formed. The local strain decreases the energy required to transform the prefusion structure to the postfusion structure [3,21]. Computational studies suggest that many of these cavities remain intact and are located at strategic positions on hinges and interfaces of the protein, where the largest amount of structural change occurs upon fusion [1,27]. The goal of the cavity-filling strategy is to remove some of the instability associated with the prefusion structure through the introduction of amino acid changes that produce residues with larger or more hydrophobic side chains, which occupy internal cavities left in the prefusion structure [2,3,28]. Importantly, the term “cavity-filling mutations” refers to a broad structure-based protein engineering strategy rather than a specific mutation set. Cavity-filling mutations involve rational amino acid substitutions designed to occupy under-packed hydrophobic cavities or internal voids that contribute to conformational instability within metastable prefusion proteins. These substitutions are designed to improve packing density and can strengthen van der Waals interactions, reduce solvent accessibility, and increase conformational stability, depending on the structural context [4]. In contrast, DS-Cav1 is a specific engineered prefusion-stabilized RSV F GP construct containing multiple stabilizing mutations, including cavity-filling substitutions, and therefore represents one example of the broader cavity-filling engineering strategy rather than defining the strategy itself. This distinction is important because stabilization by cavity-filling mutations has subsequently been applied across multiple viral vaccine platforms beyond RSV. Appropriately selected cavity-filling substitutions have been shown to improve prefusion stability in several viral systems by optimizing core packing. However, the magnitude of stabilization varies depending on protein architecture and cavity environment, as discussed in detail in the Molecular Principles of Cavity-Filling Stabilization section. Because cavity-filling mutations are often introduced outside major antigenic sites, they may preserve epitope presentation; however, the effects on antigenicity remain context dependent and require experimental validation [6,29].

The cavity-filling mutations strategy has contributed to the broader transition from empirical protein engineering toward rational, structure-guided stabilization approaches. This rational approach uses predictive computational models, molecular dynamics (MD) simulation methods, and energy-based scoring functions to identify potential sites for cavity-filling mutations and to predict which mutations most likely to be energetically favorable [2,27,30]. Improvements in algorithms such as Rosetta, FoldX, and more recent artificial intelligence (AI)-driven protein design software have improved the ability to systematically identify energetically favorable cavity-filling mutations [31].

### Scope and organization of this review

In contrast to many previously published reviews discussing multiple prefusion stabilization strategies, this review focuses specifically on cavity-filling mutations as a separate structure-based engineering approach and considers proline substitution, cysteine engineering, protomer cross-linking, and related approaches to provide structural context and as secondary or supportive mechanisms rather than the focus of this review. In addition, we examine how structural stabilization influences antigenicity and immunogenicity and discuss the integration of cavity-filling mutations with complementary stabilization approaches. Finally, we consider the broader implications of these strategies for vaccine development and structure-based antigen engineering. Recent developments further highlight the importance of integrating computational biology, structural biophysics, and rational protein engineering for next-generation vaccine development [32]. Although multiple reviews have discussed prefusion stabilization and structure-based vaccine antigen engineering, a focused synthesis specifically addressing cavity-filling mutations as a rational stabilization strategy across diverse viral fusion proteins remains limited. Therefore, this review uniquely integrates thermodynamic mechanisms underlying cavity-filling mutation-mediated metastability and their translational implications for vaccine development. By consolidating these concepts into a unified framework, this article aims to provide mechanistic insight and practical guidance for future structure-guided vaccine antigen engineering. To the best of our knowledge, this review provides one of the first comprehensive syntheses of the thermal stability of metastable states caused by cavities, computational and experimental methods for identifying cavities in proteins, application of cavity-filling mutations to different classes of viral fusion proteins, and the translationally relevant consequences of such stabilization for the generation of vaccine candidate antigens.

To create consistency within a common conceptual framework for this study, all major prefusion stabilizing methods based on structural aspects that have been described in the literature will be grouped by main molecular mechanism (Table 1). Although many different types of engineering methodologies have led to successful prefusion stabilization, it is the filling of cavities by mutation that will be reviewed as the main focus of this paper. In addition to providing background on the molecular mechanisms of cavity-filling mutations, this review discusses examples in which cavity-filling mutations are combined with complementary prefusion stabilization strategies.

**Table 1.** Conceptual classification of prefusion stabilization strategies discussed in this review

Stabilization strategy	Primary molecular mechanism	Representative examples	Role in this review
Cavity-filling mutations	Fill internal hydrophobic cavities to improve packing efficiency, increase van der Waals interactions, and stabilize the prefusion conformation	RSV F (S190F, V207L); cavity-filling mutations in viral fusion proteins	Primary focus of this review
Proline substitutions	Restrict backbone flexibility and reduce conformational entropy	SARS-CoV-2 S-2P, HexaPro	Comparative strategy
Engineered disulfide bonds	Lock flexible domains or interfaces through covalent cross-links	RSV DS-Cav1 (S155C–S290C)	Complementary stabilization strategy
Electrostatic engineering (salt bridges)	Introduce favorable electrostatic interactions to stabilize local structure	T961D and related engineered variants	Complementary strategy
Interprotomer locking	Strengthen quaternary interactions between protomers	Engineered trimer interface mutations	Comparative strategy
Trimerization domains	Promote stable trimer assembly	Foldon, GCN4	Comparative strategy

Throughout this review, references to the effects of cavity-filling mutations should be interpreted within the context of individual protein architecture, as the structural and functional consequences of these mutations vary among viral fusion proteins and require experimental validation.

## Structural Biology of Viral Fusion Proteins

### Classification and architectural principles

**Fig. 1.**
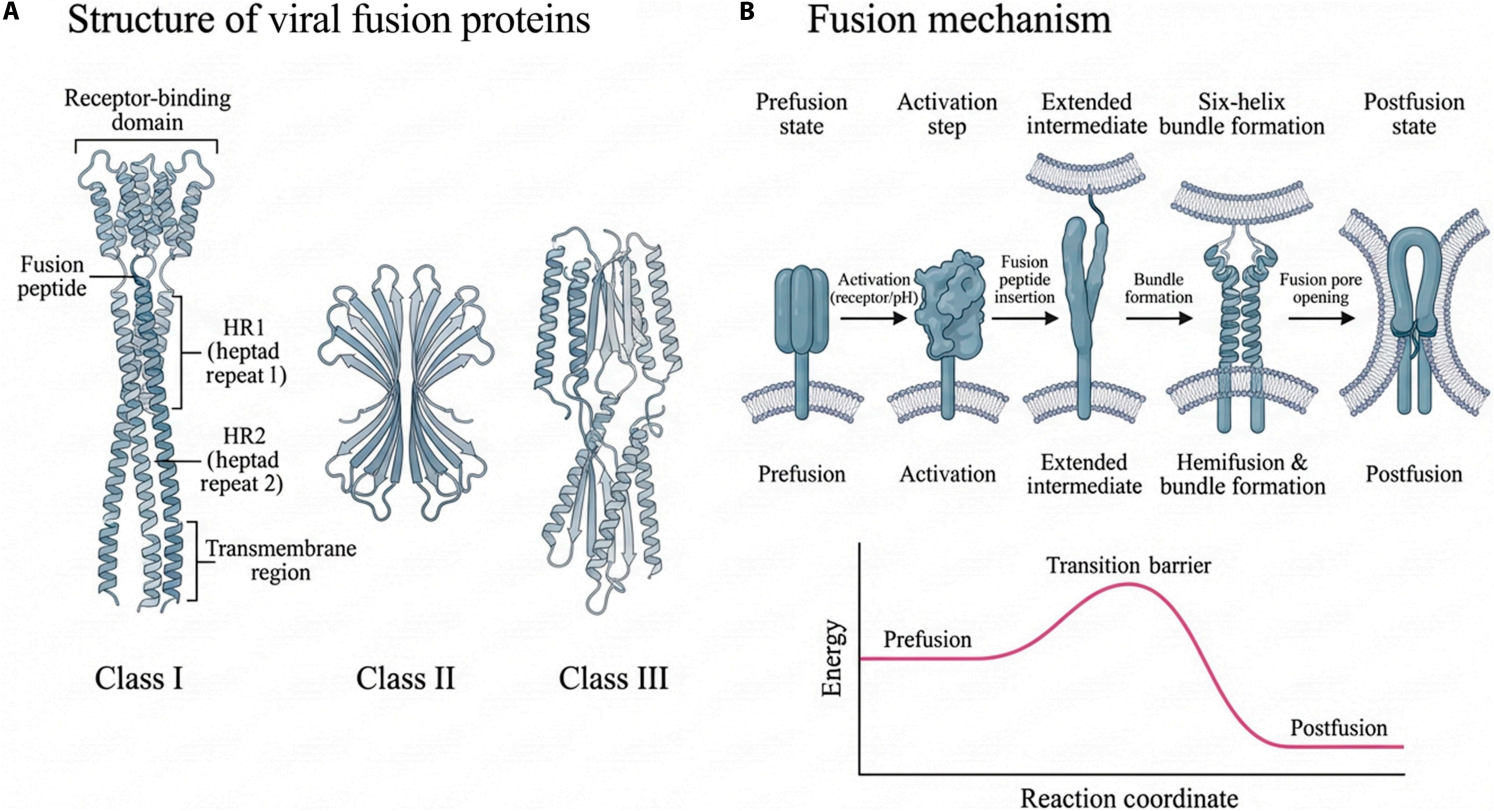
Structural classes and membrane fusion mechanisms of viral fusion proteins. (A) Structure of viral fusion proteins. Schematic depiction of the 3 principal categories of viral fusion proteins. Class I proteins possess α-helical coiled-coil structures that include a receptor-binding domain, an N-terminal fusion peptide, heptad repeat regions (HR1 and HR2), and a TM domain. Class II proteins are mostly rich in β-sheets and exist as dimers in the prefusion state, which reorganize into trimers during fusion. Class III proteins display structural characteristics of both class I and class II, incorporating core α-helices and β-sheet domains, and undergo reversible conformational alterations. (B) Fusion mechanism. The sequential stages involved in viral membrane fusion are depicted. Fusion proteins shift from a metastable prefusion state to an active state, induced by receptor contact or alterations in pH. This results in the creation of an elongated intermediate, which exposes and integrates the fusion peptide into the host membrane. The subsequent development of a 6HB (in class I proteins) or a similar refolding process brings viral and host membranes into proximity, leading to hemifusion, followed by the construction and growth of a fusion pore into the postfusion state. The lower panel illustrates the associated energy landscape, emphasizing the transition barrier between prefusion and postfusion conformations, with the postfusion state denoting a lower-energy, stable configuration. The biological significance of these structural and energy changes is that they demonstrate how the prefusion form of an envelope protein is naturally unstable and provide a basis for rational approaches toward designing vaccines that stabilize such structures in order to maintain the neutralizing epitopes.

Viral fusion proteins mediate membrane fusion, providing the mechanism for bringing together the viral and host membranes, which is necessary for the entry and infectivity of viruses [33–35]. Although the viral families vary significantly in terms of the characteristics of the fusion proteins, the proteins show similar biophysical properties and therefore can be classified into 3 structural categories based on the overall architecture of the proteins, the mechanisms of activation, and the conformational changes [35]. Viral fusion proteins are broadly classified into class I, class II, and class III categories based on their structural architecture and fusion mechanisms, as illustrated in Fig. 1A. Despite substantial differences in secondary-structure composition and activation pathways, all 3 classes utilize metastable prefusion conformations that undergo extensive structural rearrangements to drive membrane fusion. Class I fusion proteins have been the subject of extensive study and represent the primary focus of this review [36,37]. Class I fusion proteins are typically trimeric when in the prefusion state, are composed primarily of α-helical secondary structures, and include several well-studied viral GPs, such as the HA of the influenza virus, the envelope GP (Env) of HIV, the fusion (F) protein of RSV, the S GP of coronaviruses including SARS-CoV-2, the F and HN proteins of paramyxoviruses, and the GP of Ebola virus [38–42]. These proteins are highly metastable and have evolved a standard functional architecture that supports the rapid conversion from the prefusion to the postfusion state [33,35]. The typical architecture of class I fusion proteins is modular, which reflects their dual role in the binding of host cell receptors or attachment factors and the subsequent fusion of membranes. Each protomer of a class I fusion protein usually comprises a receptor-binding subunit (or domain) and a fusion subunit. The receptor-binding subunit mediates the interaction with host cell receptors or attachment factors.

The fusion subunit includes the fusion peptide (FP), an extremely short hydrophobic region of the GP that is embedded in the target membrane following activation [43]. The fusion subunit also contains heptad repeat regions (HR1 and HR2), which form coiled-coil motifs that support both the stability of the prefusion trimer and the refolding of the postfusion GP [36,37]. The transmembrane domain (TM) anchors each GP protomer to the viral envelope, while a cytoplasmic tail may mediate associations with the viral matrix or nucleocapsid. In the prefusion state, the FP is completely buried within the central cavity of the trimer and inaccessible to solvent, preventing early activation [6,39]. This prefusion state of the class I fusion proteins represents a high-energy metastable state that is primed for conversion to the low-energy postfusion state following activation.

The remaining classes exhibit distinct structural features, some of which include specific structural features. For example, class II fusion proteins, which are found in flaviviruses and alphaviruses, are primarily composed of β sheets and exist as dimers before fusion but transform into trimers after fusion [34,35]. Class III fusion proteins, which include the vesicular stomatitis virus (VSV) G protein and the herpesvirus gB protein, contain both α helices and β sheets and operate as trimers that are activated through interactions with receptors or a decrease in pH [35]. Regardless of their structural differences, all viral fusion proteins share a common mechanistic strategy: They store potential energy in a metastable prefusion state that is released upon activation to induce membrane fusion [33,34]. Viral fusion proteins are classified into class I, class II, and class III according to their structural architecture and fusion mechanisms (Fig. 1).

### The prefusion-to-postfusion transition: Structural and energetic basis of conformational rearrangement

Building upon the overview presented in The challenge of viral fusion proteins section, this section examines the molecular events governing the prefusion-to-postfusion transition, during which viral fusion proteins undergo large-scale refolding into a thermodynamically favorable postfusion state. This transformation involves considerable refolding, movement of domains, and reorganization of secondary and tertiary structural features, often at distances greater than 100 Å [44,45]. The transformation results in a highly stable postfusion conformation that is virtually irreversible under physiological conditions, and is generally accompanied by a large loss in free energy (Δ*G* ≈ −40 to −80 kcal/mol for the trimeric assembly) [46]. The relatively large negative free energy change (−Δ*G*) indicates the release of stored potential energy that drives membrane fusion [35,37,45].

The activation of the fusion sequence begins with some specific triggering event that can differ among various viral families [47]. Acidification of the endosome following viral entry activates influenza HA. Coronaviruses require receptor binding (e.g., ACE2 engagement), as well as proteolytic cleavage at the S2′ site, to activate the transition [48]. In HIV Env and RSV F, receptor binding and either elevated temperature or pH can stimulate the fusion sequence [49–51]. These triggering events disrupt essential interdomain contacts within the prefusional state of the protein and allow the FP to extend toward the host membrane.

The fusion sequence then goes through several different conformational stages until it reaches its postfusion form. In the first step of this process, the FP (or fusion loop) is extended toward the host cell membrane, forming an intermediate prehairpin that temporarily bridges both the viral and host cell membranes [33]. During this time, the HR1 regions form a single, large trimeric helical bundle (coiled-coil), with all 3 monomers extending along their entire length from each end of the trimer.

Subsequently, the HR2 regions fold back onto the HR1 core and bind to the HR1 regions to form the 6-helix bundle (6HB) structure [52]. The rod-like postfusional 6HB assembly positions the viral and target membranes closely together to facilitate lipid mixing and membrane merger. The refolding process is not only a structural rearrangement, but it is also a mechanochemical process. The energy released during the formation of the 6HB and other structural changes is used directly to perform mechanical work that allows the energy barrier to membrane fusion to be overcome. Once the postfusional conformation has been achieved, the system becomes stable in a low-energy configuration, and thus, the process is effectively irreversible.

The conversion from the prefusion to the postfusion forms illustrates the remodeling of the energy landscape from a thermodynamic standpoint, as summarized in Fig. 1. The prefusional state is located at a local minimum, separate from the postfusional state by an energy barrier that is overcome when the fusion sequence is activated. The high-energy prefusional state is inherently unstable and will undergo spontaneous refolding, which is unfavorable for the production of recombinant antigens, but necessary for the productive infection of viruses.

### Metastability and its molecular determinants

The metastable state of viral prefusion fusion proteins arises from a balance between stability and reactivity created through an array of molecular characteristics. Three major structural features contribute to this unique combination of metastability: internal cavities, electrostatic frustration, and conformational strain [53].

#### Internal cavities and other packing defects

As established in the Cavity-mediated instability and the rationale for cavity-filling section, internal cavities are an important contributor to prefusion metastability. Here, packing defects, true internal cavities, and transient cavities are defined as related but distinct structural features. Packing defects include loosely packed hydrophobic regions, small internal voids, and suboptimal van der Waals contacts. True internal cavities are solvent-inaccessible voids that persist within the protein core, whereas transient cavities arise dynamically during normal protein motion and do not persist over time. Therefore, while all internal cavities are defects in packing efficiency, not all packaging defects create identifiable cavities.

Consequently, mutations introducing larger or more polar side chains to fill these voids can enhance structural stability when the substituted residue is compatible with the surrounding protein environment [54].

#### Electrostatic frustration and charge imbalance

Electrostatic frustration, another important contributor to metastability, arises from charged or polar residues being placed in an environment where they are unable to make stable electrostatic interactions because they are located in a hydrophobic environment or near like-charged residues [55,56]. Lysines, arginines, and aspartates in viral fusion proteins can be located on the surface of the prefusion protein or buried inside [51,57]. The presence of these residues creates an environment of internal stress and thus contributes to the structural instability of the prefusion protein [24]. However, as the protein undergoes the conformational change, many of these buried residues will become exposed to solvent and/or form new salt bridges that contribute to the stability of the protein [24,58]. Thus, the frustration created by electrostatic interactions acts as a molecular reservoir of conformational energy, storing conformational energy that is then rapidly released during the conformational change. Although this frustration contributes to rapid triggering, it also renders the prefusion protein inherently unstable during recombinant expression or purification. Therefore, strategic substitutions that relieve buried charges and/or create new complementary interactions to those existing in the wild-type protein can help stabilize the prefusion state, provided that they do not impede the ability of the protein to bind its receptor and activate fusion.

#### Backbone strain and conformational preorganization

The polypeptide backbone of viral fusion proteins also contributes to metastability by creating localized strains in loops, twists, and hinge points that connect the major structural domains (e.g., the FP and HR1 domain). Frequently, these strained regions align with the “hinge” regions that move during the prefusion-to-postfusion transition. For example, in the case of influenza HA and the SARS-CoV-2 S protein, high B-factor values are observed and low hydrogen bonding densities in the hinge areas adjacent to the FP and HR1 domain, indicating increased dynamic instability. By preparing the protein for refolding through the creation of these strained regions, backbone strain reduces the activation energy required for the protein to undergo the conformational change upon activation. Nevertheless, this flexibility is detrimental to the maintenance of the structure during recombinant antigen production since the protein may partially unfold spontaneously, regardless of the presence of physiological stimuli [23]. The use of proline substitutions has been successful in limiting the range of motion of the backbone due to the limited number of possible torsion angles available to the polypeptide backbone, thereby reducing the negative impact caused by backbone strain on the stability of the protein [59].

### Integrative perspective

Structural stability and conformational flexibility are both essential for viral fusion proteins to undergo rapid membrane fusion while retaining their ability to enter host cells. As such, understanding how prefusion stability is determined has allowed researchers to develop methods based on rational design to preserve specific conformations of antigens for vaccines. One such strategy for this purpose is cavity-filling, which has been shown in several viral fusion proteins to improve prefusion stability and, in many reported systems, preserve antigenic and immunogenic properties, although outcomes remain protein-dependent. In addition to the mechanisms and applications described below, the following sections describe stabilization by cavity-filling mutations in viral fusion protein engineering.

## Molecular Principles of Cavity-Filling Stabilization

### Thermodynamic basis

Protein stability is governed by the balance between favorable enthalpic interactions and entropic contributions, both of which determine the free energy difference between the folded and unfolded states [60,61]. Each structural feature that decreases the number or strength of the favorable enthalpic interactions will result in a measurable thermodynamic cost. Among the most destabilizing structural features are internal cavities, which represent regions of incomplete van der Waals packing within the hydrophobic core of proteins [62]. The free energy costs of cavities are generally proportional to their size: Large cavities have a greater destabilizing effect than small ones, and even very small cavities may be used as nucleation sites for localized unfolding. Published studies have reported stabilization energies of approximately 3 to 15 kcal/mol per mutation, although these values depend on cavity geometry, residue identity, and experimental conditions [54,63].

**Fig. 2.**
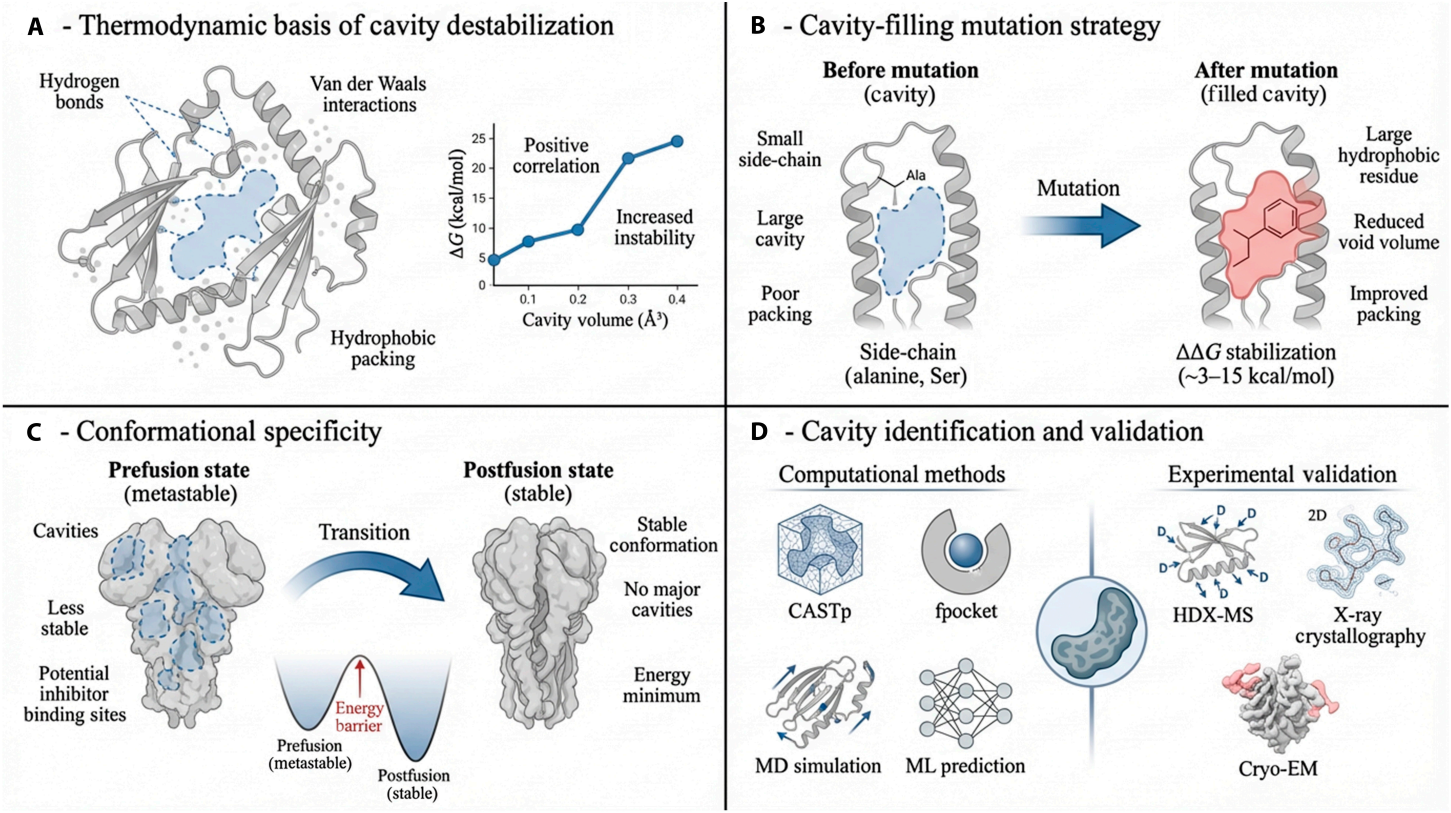
Molecular principles and identification of cavity-filling stabilization in proteins. (A) The overall stability of proteins exists in a delicate equilibrium between the favorable contributions from enthalpy due to attractive interatomic forces and unfavorable contributions from entropy resulting from restriction of the possible configurations of the unfolded polypeptide chain. Regions of internal cavitation exist throughout the hydrophobic core of many globular proteins. Cavitation results in a loss of van der Waals contacts among nonpolar residues and an increase in the free energy of the polypeptide. These areas may also serve as focal points for local unfolding events. (B) Substitution of smaller residues, such as alanine or serine, with larger hydrophobic residues, including leucine or phenylalanine, at positions that correspond to internal cavities may improve the thermodynamic stability of the protein. This substitution can improve packing efficiency within the hydrophobic core, decrease void volume, and restrict solvent access into the core—all factors that contribute to decreased conformational freedom. (C) For viral fusion GPs, which are metastably stabilized in their prefusion states, cavity-filling mutations have been shown to improve prefusion stability in several viral systems. By selectively substituting residues at sites that are specific to the prefusion state, these mutations may create higher barriers to conformational transition and, in favorable structural contexts, may help preserve antigenic determinants relevant for vaccine-induced immunity. (D) To identify and validate candidate cavities for stabilization using this approach requires both extensive use of theoretical models and experimental characterization of protein structure and function. Computational modeling (e.g., MD), geometric analysis, and machine learning prediction techniques are used to model the behavior of predicted cavities. Experimental verification of these predictions uses a variety of biophysical techniques, including x-ray crystallography, cryo-electron microscopy (cryo-EM), and hydrogen/deuterium exchange mass spectrometry (HDX-MS) to measure changes in protein packing and dynamics. The biological significance of all 3 panels is that they represent the structural justification for the stabilization provided through cavity-filling mutations. Internal cavities, along with other types of packing defects, are detrimental to packing efficiency as well as contributing to the inherent metastability of prefusion forms of viral fusion proteins. In theory, rationally selected cavity-filling mutations may improve packing within the hydrophobic core, eliminate or at least minimize the volume of space (void) associated with undesirable packing defects, and therefore may enhance the free energy barrier against nonspecifically induced transition from the prefusion form to the postfusion form. Furthermore, combining predictive modeling techniques with empirical verification provides a methodical means of identifying those mutations that would provide enhanced stability of the prefusion structure while maintaining those epitope characteristics necessary for successful vaccine development.

Cavity-filling mutations typically replace short side chains (e.g., alanine, serine, or threonine) with larger hydrophobic residues such as leucine, isoleucine, phenylalanine, or tryptophan [25,54]. These substitutions can increase van der Waals interactions, reduce internal void volume, and reduce the presence of energetically unfavorable water molecules. They may also influence thermodynamic stability by reducing the conformational entropy of the native structure. Loosely packed structures typically exhibit increased side-chain motion and localized conformational fluctuations. Appropriately placed cavity-filling mutations can restrict these motions by creating additional complementary van der Waals contacts, thereby reducing the number of accessible side-chain conformations. The decreased conformational entropy associated with cavity-filling mutations typically has an entropic cost but may be compensated for by an increase in enthalpy due to the formation of better van der Waals contacts. Therefore, the overall thermodynamic effect on protein stability depends upon a balance of the enthalpic and entropic costs based on the specific structural context of each protein. When introduced into appropriately selected cavities, these mutations can improve packing efficiency, reduce local conformational flexibility, and enhance folding stability. This strategy has demonstrated utility in several reported viral GP systems. Many prefusion conformations contain cavities that arise due to the geometric limitations of the metastable conformation of the protein and disappear when the protein refolds into the postfusion conformation [64]. Accordingly, filling appropriately selected prefusion-specific cavities may increase the energetic barrier to conformational transition without stabilizing the postfusion conformation. In many reported cases, cavity-filling mutations preferentially stabilize the prefusion conformation and contribute to preservation of antigenically relevant structural features, although outcomes vary among viral systems. Identifying the cavities that contribute most to the metastability of the prefusion conformation requires detailed structural and energetic analysis. A variety of structural factors determine the suitability of a given cavity to be targeted by stabilizing mutations [65]. Therefore, cavities that are buried within the prefusion trimer’s densely packed hydrophobic core are generally expected to have a greater impact than surface- or partially solvent-accessible cavities. Similarly, the geometry and size of a cavity must allow for the placement of a larger amino acid residue without creating steric clashes or disrupting the surrounding secondary structure. Studies have shown empirically that optimal cavity volumes for single-residue filling are usually between 30 and 120 Å and correspond to the volume differences between small polar and large hydrophobic side chains [66]. Cavities that are too large may require one or several additional coordinated mutations or cooperative rearrangement of packing interactions to achieve a level of stabilization. Additionally, conformational specificity is important for stabilizing the protein structure since it requires identifying cavities that are unique to the prefusion conformation rather than those that are also present in the postfusion conformation. Thus, high-resolution comparative structural analysis of both the prefusion and postfusion structures of the protein is necessary to identify regions where cavity-filling mutations can increase the energetic difference between the 2 states, as illustrated in Fig. 2. The thermodynamic consequences of cavity formation and the rationale for stabilization by cavity-filling mutations are summarized in Fig. 2A to C.

### Cavity identification and characterization

#### Computational approaches

The use of computational algorithms along with structural bioinformatics has made cavity analysis predictive and quantitative. Algorithms such as CASTp [66], HOLLOW [67], CAVER [68], and fpocket [69] allow us to quantitatively identify all cavities present in a protein structure. All of these algorithms utilize geometric concepts, specifically derived from α-shape theory, Voronoi tessellations, and grid-based probe insertions, to define the interior cavities and how accessible they are, as depicted in the lower panel of Fig. 2. Representative computational and experimental approaches used for cavity identification and validation are shown in Fig. 2D.

In addition to static structural analysis, MD simulations reveal how cavities appear, disappear, or change size during protein motion. Transient cavities that form and dissolve as a protein undergoes fluctuations in its motion typically occur at locations where the protein begins to unfold. The ability to sample conformational space beyond what is represented by experimental structures is facilitated by metadynamics, accelerated MD, and Gaussian accelerated MD. As an example, these simulations can find cavities that become larger as the protein undergoes the early stages of the prefusion-to-postfusion transition and can highlight potential “structurally vulnerable regions” for the stabilization of the protein. Recent advances in the development of machine learning-based models now provide ways to improve upon traditional geometric methods used to predict cavities. These machine learning models, which include structural descriptors, energy terms, and residue-environment terms, are used to predict which cavities are most likely to destabilize the prefusion state. In addition to providing tools to rapidly identify successful cavity-filling mutations in a variety of viral systems, these models create a framework for deep learning-based approaches to protein design. Emerging computational frameworks integrating machine learning and structural modeling further improve the prediction of stabilizing mutations and protein engineering outcomes [70].

#### Experimental approaches

Experimentation provides the means to validate computational predictions and assess local flexibility and the interior protein packing [71]. Several high-resolution biophysical techniques can provide complementary views of this process, as described below. Hydrogen-deuterium exchange mass spectrometry (HDX-MS) measures the rate of hydrogen-deuterium exchange of the backbone amide hydrogens of a protein in solution [72]. Regions of loosely packed interior or new cavity formation show increased rates of exchange, and thus represent sites of structural liability that could potentially be stabilized through cavity-filling mutations. X-ray crystallography is still the leading method for directly visualizing cavities. Unoccupied cavities are frequently visible as low-density regions or as regions of structurally ordered solvent within otherwise predominantly hydrophobic interiors, provided that the resolution of the crystal structure is greater than 2 Å. Discontinuous or weak electron density associated with side chain atoms typically indicates the presence of an interior void or structural heterogeneity. Amidst this, the cryo-EM is increasingly capable of delivering sufficient detail to visualize interior cavities, particularly when coupled with the use of local resolution mapping and computational refinement [73]. Regions of flexible density in cryo-EM density maps often correspond to cavity-containing regions that exhibit conformational changes during fusion. The corresponding experimental approach is illustrated in Fig. 2.

Although cavity-filling mutations have successfully stabilized multiple viral fusion proteins, their effects are not universally predictable. The impact of a given substitution depends on factors including cavity geometry, local packing interactions, conformational dynamics, and epitope architecture. In some cases, mutations may fail to improve stability or may adversely affect protein expression, folding, or antigenicity. Consequently, computational predictions should be interpreted as hypotheses requiring experimental validation through structural, biophysical, and immunological characterization. Importantly, not all cavity-filling mutations identified through computational analysis prove beneficial experimentally. For example, during the development of RSV F (DS-Cav1), as well as subsequent stabilization of coronavirus S GPs, multiple cavity-filling substitution candidates predicted by computation were subsequently evaluated using experimental methods; however, fewer than half of these candidates resulted in significant improvement of the proteins’ prefusion stability. In addition, many potential substitutions were eliminated from further consideration because they failed to produce an increase in protein expression, folding, thermostability, or antigenicity. These results illustrate that mutations designed to fill cavities can be extremely context-dependent and that successful stabilization of such structures is contingent on integrating both predictive modeling of structure and computational predictions with experimental verification. During the development, several stabilized viral fusion proteins, including RSV F and coronavirus S GPs, were evaluated, but only a subset produced improvements in protein expression, thermostability, or antigenicity, while others were discarded because of neutral or unfavorable effects on folding or structural integrity [4,23–25]. These observations emphasize that cavity-filling mutations are inherently context-dependent and require iterative experimental optimization.

## Design and Implementation of Cavity-Filling Mutations

**Fig. 3.**
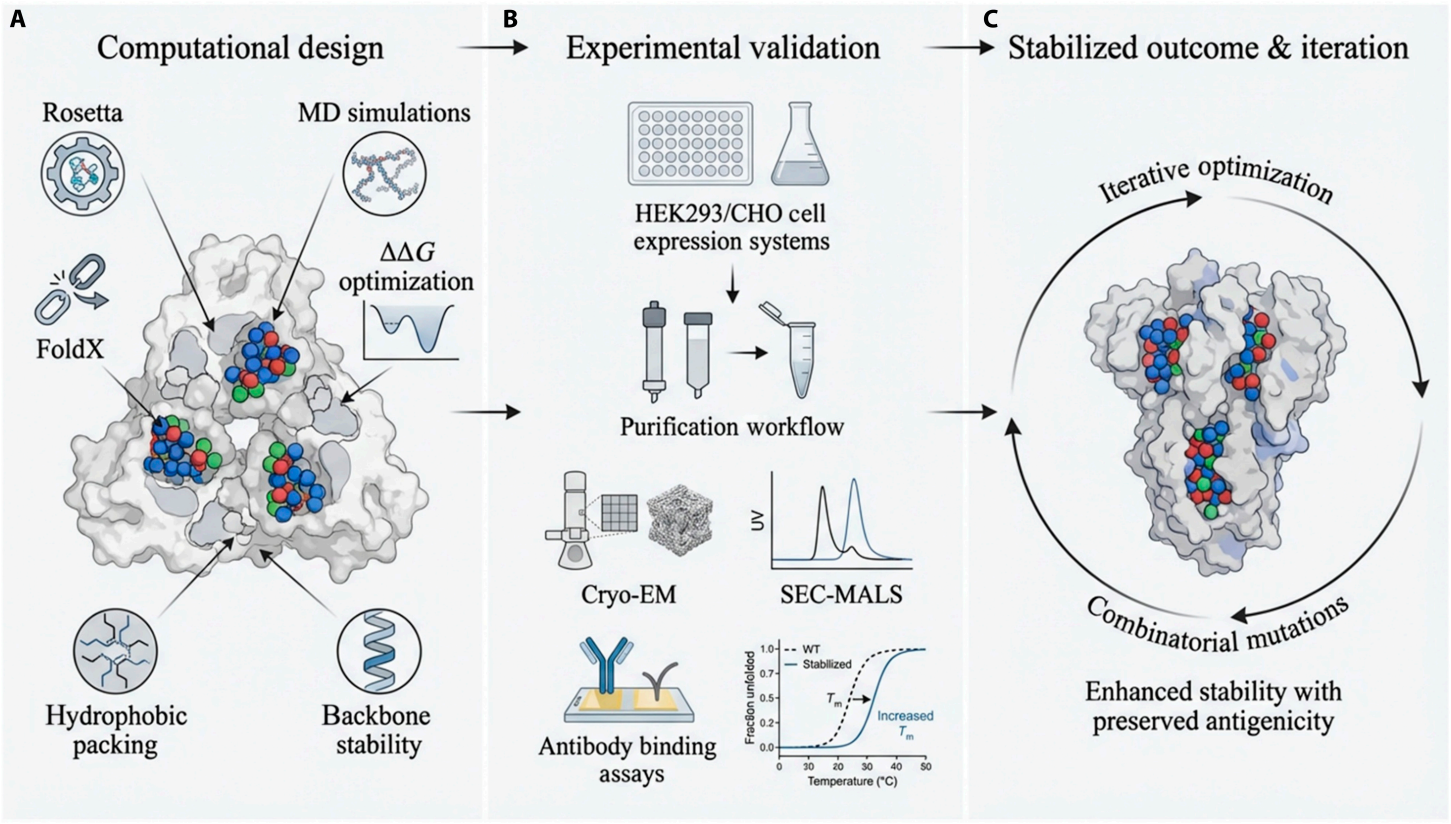
Computational design, experimental validation, and iterative optimization of prefusion-stabilized viral fusion proteins. (A) Computational design: Computationally based approaches that utilize structural information guide the identification of substitutions within a viral fusion protein that will stabilize it in its prefusion form. These include prediction tools (Rosetta & FoldX), which compute ΔΔ*G* values from substitution mutations that improve the hydrophobic core-packing, cavity-filling mutations, and backbone stabilization; molecular dynamic simulations measure the long-term structural stability and conformational flexibility of the mutated viral fusion proteins. (B) Experimental verification: Each designed variant is produced by expressing it in mammalian cells (HEK293/CHO), purified and analyzed using cryo-EM and SEC-MALS. In addition, each variant is tested using an antibody binding assay to verify its ability to bind antibodies specific to the viral fusion protein; *T*_m_ determinations are made to determine if the substitutions have stabilized the variant relative to its wild-type counterpart. (C) Iterative refining: Substitutions identified during computational design and verified experimentally are iteratively combined and refined through multiple computational–experimental cycles, resulting in a single, extremely stable, prefusion immunogen that retains all critical neutralizing epitopes essential to vaccine development based upon structural information. Biological significance: This protocol demonstrates how the predictive tools of molecular modeling, the experimental techniques of structural biology, and experimental analysis are combined as part of a cyclical design process to predict mutations that will stabilize the prefusion state while conserving structural integrity and immunogenic potential.

The rational design of cavity-filling mutations requires integration of structural biology, computational modeling, molecular simulations, and experimental validation to identify substitutions that may enhance prefusion stability while preserving antigenic integrity. The central goal is to identify amino acid substitutions that eliminate energetically unfavorable cavities without compromising protein function. It appears that many of these cavities are thought to contribute to the “breathing” mechanism, which means that they are generally unstable and tend to spontaneously adopt their postfusion configuration. Large hydrophobic amino acid side chains (e.g., valine, leucine, isoleucine, phenylalanine, and methionine) may promote more efficient hydrophobic packing and van der Waals interactions within the protein core, potentially reducing solvent penetration. Several studies have reported that cavity-filling mutations may lead to reduced conformational flexibility and greater levels of recombinant protein expression than unmutated counterparts. These benefits are often realized when combined with other approaches such as proline substitutions, formation of engineered disulfide bridges, helix capping mutations, and interprotomer lock mutations. Biochemical and structural data suggest that a well-designed cavity-filling mutation strategy may help preserve important neutralizing epitopes while preventing excessive contact with overly flexible or unstable non-neutralizing epitopes. The application of this concept has recently been extended to additional class I viral fusion proteins, including those from RSV, 2 strains of influenza virus, HIV-1, coronaviruses, and paramyxoviruses. As previously discussed, these principles have contributed to the development of rationally designed vaccine antigens and second-generation immunogens across several viral systems. A schematic illustration and general overview of the workflow for the computational design, experimental validation, and iterative optimization of cavity-filling mutations are shown in Fig. 3.

### Strategies for computational design

As discussed in the Molecular Principles of Cavity-Filling Stabilization section, the goal of computational design is to find amino acid replacements that maximize both the packing efficiency of the cavity and structural integrity. In this section, we will focus on the computational tools and protein engineering methodologies used to computationally design candidate mutations for subsequent laboratory-based experimentation. The Rosetta software suite has emerged as one of the most commonly utilized platforms for rationally designing proteins for stabilization [74]. It provides atomistic models to predict the substitution effects of individual amino acids on areas of interest and the total energy of the folded structure. As a result, the Rosetta-specified fixed-backbone model provides a robust method for assessing changes in the residue lining the cavity. The protein native structure is preserved by improving the burial of residues in the hydrophobic core without damaging the main chain with structural changes. However, this modified design might not always be sufficient under certain conditions and may require moderate backbone adjustments. Employing a flexible backbone strategy can help fine-tune mutations, allowing for slight modifications in shielding. In addition to Rosetta, FoldX is another rapid and semi-empirical method for estimating the thermodynamic effect of single-point mutations (ΔΔ*G*) based upon knowledge-based potentials. Consequently, FoldX complements Rosetta by providing rapid estimation of mutation-induced ΔΔ*G* values. Its computational efficiency enables high-throughput screening that, in turn, limits the mutation types that can be more correctly modeled. Such analysis is crucial for computational functional group design and mutation enumeration, especially when in silico confirmation of the changes is needed to fill the cavities. In cavity-filling mutation application, FoldX has proven particularly effective in the enhancement of residue substitutions that improve the hydrophobic packing, decrease the density of internal voids, and increase the steric complementarity of the protein core. Advances in the AI (machine learning)-driven protein design field have further enhanced the ability of point mutations to be predictably introduced. By utilizing machine learning tools such as ProteinMPNN [75] and AlphaFold, along with the concept of residue co-evolution, it becomes feasible to forecast modifications that are thoughtfully integrated to improve the functional display of secondary structures. In addition, ProteinMPNN and RFdiffusion [76] have significantly expanded the capabilities of rational immunogen engineering. ProteinMPNN utilizes graph neural network-based sequence design to optimize amino acid packing and structural compatibility in protein backbones, enabling rapid identification of stabilizing mutations in viral GPs. RFdiffusion applies diffusion-based generative modeling to create novel protein architectures and conformationally stabilized scaffolds with high structural fidelity. The methods described above augment current traditional Rosetta- and MD-based methods through the ability to rapidly and efficiently explore large sequences of structure space and accelerate the design of prefusion-stabilized antigen. In addition, using the sequencing methods described to analyze alignment of sequences, patterns of co-evolution, and kinetics may help to identify favorable mutation candidates that are less than obvious due to their low energy scores. The use of cavity search algorithms such as CASTp, Fpocket, and CAVER allows examination of internal spaces, finding hidden pocket structures, and identifying hydrophobic cavities within the interior surface of proteins prior to any modification. Furthermore, MD simulations in conjunction with free energy perturbation (FEP) provide a quantitative analysis of thermodynamic contributions and motion-induced displacements created by cavity-filling mutations. Although it takes significant computational resources to utilize these methods, they provide insight into how solvent interactions, neighboring side chains, and reformation of adjacent side chains influence both dynamic behavior and functional activity in proteins. To understand whether creating larger residues will stabilize in real-world environments, MD simulations are used. It is unclear if increasing residue size would cause instability, complicating protein assembly, thereby negatively impacting protein function. Furthermore, recent trends include applying enhanced sampling methods, such as replica exchange MD and metadynamics, to assess changes to long-term stability caused by active site cavity creation by introducing multiple mutations in enzyme engineering applications.

#### Emerging AI-driven protein design and high-throughput validation approaches

Recent advancements in artificial intelligence (AI) have had a significant impact on the field of protein engineering and the design of vaccines based on protein structures. The use of deep learning technologies like AlphaFold and AlphaFold3 [77] has substantially improved the prediction of accurate protein structures and the identification of areas of proteins where cavities exist. In addition to the improvements seen in predicting protein structures, numerous additional protein structure prediction technologies, including ESMFold [78,79] and other protein language models, have been developed from their amino acid sequences. These types of technologies have enabled high-throughput screening of possible antigen candidates.

In addition to the improvement in the ability to predict protein structures, there has also been an emergence of rational antigen engineering technologies using generative protein design frameworks. A number of different technologies have been developed to apply machine learning algorithms and generative modeling to engineer proteins. Examples include ProteinMPNN, which applies graph neural networks to identify optimal amino acid sequences that preserve the structural characteristics required for a particular antigen. Additionally, technologies such as RFdiffusion utilize diffusion-based generative modeling to develop new protein architectures and interfaces to provide the opportunity to generate novel stabilized immunogen and engineered protein assemblies. Furthermore, emerging technology platforms such as Boltz-1 [80] and similar AI-driven design frameworks continue to combine the capabilities of structure prediction, amino acid sequence optimization, and protein stability assessments into integrated workflows.

High-throughput experimental methods, including deep mutational scanning, saturation mutagenesis, and stability profiling, have greatly accelerated vaccine antigen optimization by enabling thousands of variants to be evaluated simultaneously. This enables researchers to generate vast amounts of fitness landscape data to determine which substitutions enhance or diminish the stability of their vaccine antigens. By integrating this type of experimental data with machine learning algorithms, it is expected that researchers will be able to develop predictive accuracy of their rational antigen engineering designs. Collectively, these advances are expected to facilitate increasingly predictive and scalable protein engineering workflows.

Despite these advancements, existing AI-driven protein design methodologies exhibit several significant limitations. Most models are primarily optimized for predicting protein structure or sequence compatibility, rather than for biological function, immunogenicity, antigenic epitope preservation, long-term conformational dynamics, or manufacturability. The predictive performance may vary among different viral families due to the fact that many algorithms have been developed using datasets that do not adequately capture the structural diversity of viral fusion proteins. Thus, computational predictions ought to be viewed as tools for generating hypotheses rather than as conclusive answers. Candidate designs necessitate thorough experimental validation via structural analyses (cryo-EM or x-ray crystallography), biophysical characterization [differential scanning calorimetry (DSC), differential scanning fluorometry (DSF), size-exclusion chromatography coupled with multi-angle light scattering (SEC-MALS)], antigenicity assays, and immunogenicity studies to ensure their appropriateness for vaccine development.

### Experimental validation process

The process for experimental validation is described in Table 2, where initial steps include generating in silico predictions to identify potential beneficial mutations that could be added to improve the stability of an antigenic protein. Once these candidates have been determined, they undergo a series of experiments designed to assess their impact on the structural properties of the protein. An important first step is to express the protein at high levels from appropriate eukaryotic systems [e.g., human embryonic kidney 293 (HEK293), Chinese hamster ovary (CHO), and insect cells], such as those used for biologics production [81]. The next step in conformational analysis of the antigen is to use either negative stain EM or cryo-EM to determine the overall shape of the molecule, and to assess whether the antibody-binding sites for prefusion-specific monoclonal antibodies remain intact. Finally, additional experimental validation is required to provide definitive proof of structural integrity, antigenicity, and function of engineered antigens [82].

**Table 2.** Computational and AI-driven platforms for cavity-filling mutation design and vaccine antigen engineering

Tool/platform	Category	Primary purpose	Major computational function	Application in vaccine antigen engineering	Advantages	Limitations
Rosetta	Physics-based	Protein design	Energy minimization, mutation design	Cavity-filling mutations identification	Highly accurate	Computationally intensive
FoldX	Energy-based	Stability prediction	ΔΔ*G* calculation	Rapid mutation screening	Fast and simple	Lower structural detail
Molecular dynamics	Simulation	Dynamic stability analysis	Conformational sampling	Validation of stabilizing mutations	Captures protein motions	High computational cost
AlphaFold3	Deep learning	Structure prediction	Atomic-level structural modeling	Identification of cavity regions	High prediction accuracy	Limited direct design capability
ESMFold	Protein language model	Structure prediction	Sequence-to-structure prediction	Large-scale antigen screening	Extremely rapid	Less detailed than MD
ProteinMPNN	Generative AI	Sequence optimization	Structure-conditioned sequence design	Stability-enhancing mutation design	Excellent sequence engineering	Requires known structure
RFdiffusion	Generative AI	De novo protein design	Diffusion-based structure generation	Novel immunogen design	Creates new scaffolds	Computational complexity
Deep mutational scanning (DMS)	Experimental	Variant validation	Large-scale functional screening	Identification of stabilizing mutations	High-throughput validation	Requires experimental infrastructure

Another important step is thermal stability testing. This is determined by DSC. DSC gives an exact measure of the melting point (*T*_m_), which serves as a good indicator of increased stability. However, DSF offers a quicker method of screening large numbers of mutants. In several reported studies, increases in *T*_m_ of approximately 5 to 10 °C have been associated with successful prefusion stabilization, although the magnitude of improvement varies among proteins and experimental conditions [23,83]. Finally, studies of immunogenicity are generally conducted in small animal models and evaluate whether the modified forms of the virus induce stronger neutralizing antibody responses than their original counterparts. Iterative optimization: In most cases, it takes more than one cycle of modifications to reach an optimal solution. Therefore, iterative optimization is a common procedure that simultaneously may improve both the antigenicity and the stability of a given structure. Mutations that fill cavities within proteins can sometimes have synergistic effects when combined together. Therefore, combinatorial testing of different amino acids that replace those in a cavity is very common. Additionally, stiffening or introducing too many mutations near immunodominant epitopes may also reduce the antigen’s ability to stimulate strong immune responses. As a result, there must be a delicate balance between maximizing the stability and antigenic fidelity of a vaccine candidate while minimizing the number of mutations required to achieve this goal.

To provide a broader perspective on the application of cavity-filling mutations and related prefusion stabilization strategies across different viral systems, a comparative summary of representative examples is presented in Table 3. The quantitative values summarized in Table 3 are representative examples reported in individual studies. Since experimental systems, protein architectures, expression platforms, and analytical methods differ substantially among viral families, these values should be interpreted as illustrative rather than directly comparable or universally applicable.

**Table 3.** Representative examples of cavity-filling and related prefusion stabilization strategies reported in independent studies. Quantitative values (e.g., Δ*T*_m_, expression levels, and immunogenicity) are derived from individual publications performed under different experimental conditions and should not be interpreted as direct cross-study comparisons.

Virus	Fusion protein	Stabilization strategy	Representative mutation(s)	Structural outcome	Vaccine/application
RSV	F	Cavity-filling + disulfide	S190F, V207L, S155C/S290C	Stable prefusion DS-Cav1	Abrysvo
SARS-CoV-2	Spike	Cavity-filling + Proline	2P, HexaPro, VFLIP	Increased stability and expression	COVID-19 vaccines
Influenza	HA	Hydrophobic core optimization	Multiple cavity-filling mutations	Enhanced prefusion stability	Universal influenza vaccine candidates
Ebola	GP	Structure-guided stabilization	Core-packing mutations	Improved trimer stability	Vaccine antigen development
Nipah	F	Prefusion stabilization	Cavity-filling mutations and disulfide strategies	Preserved neutralizing epitopes	Experimental vaccine candidates

## Case Studies: Successful Applications across Viral Families

This section of case studies describes a few representative viral fusion proteins where filling cavities with mutations have helped stabilize the prefusion state. As mentioned above, often the most effective way to develop an immunogen is by combining multiple approaches (cavity-filling mutations; proline substitutions; engineered disulfide bonds; interprotomer lock; trimerization domain); therefore, these cases should be viewed as total stabilization methods and not just cavity-filling mutation methods. Although additional stabilization mechanisms will be discussed for the purposes of understanding structural and functional background information, each case study will focus on how cavity-filling mutations contribute to stabilizing the prefusion state.

### Respiratory syncytial virus

The RSV F GP is among the most well-studied examples of how prefusion stabilization has been achieved through structural guidance. Furthermore, it represents how structure-based stabilization may provide a basis for understanding the role of cavity-filling mutations within a multi-component stabilization approach. While the DS-Cav1 immunogen includes both cavity-filling mutations along with stabilized disulfides and additional stabilization components, the identification of cavity-associated instability in RSV F provided an important conceptual foundation. One consistent impediment to successful RSV vaccine development has been the ability to capture and maintain the prefusion conformation, since the prefusion form exposes the essential antigenic site Ø, a major target of potent prefusion-specific neutralizing antibodies such as D25 and AM22.

**Fig. 4.**
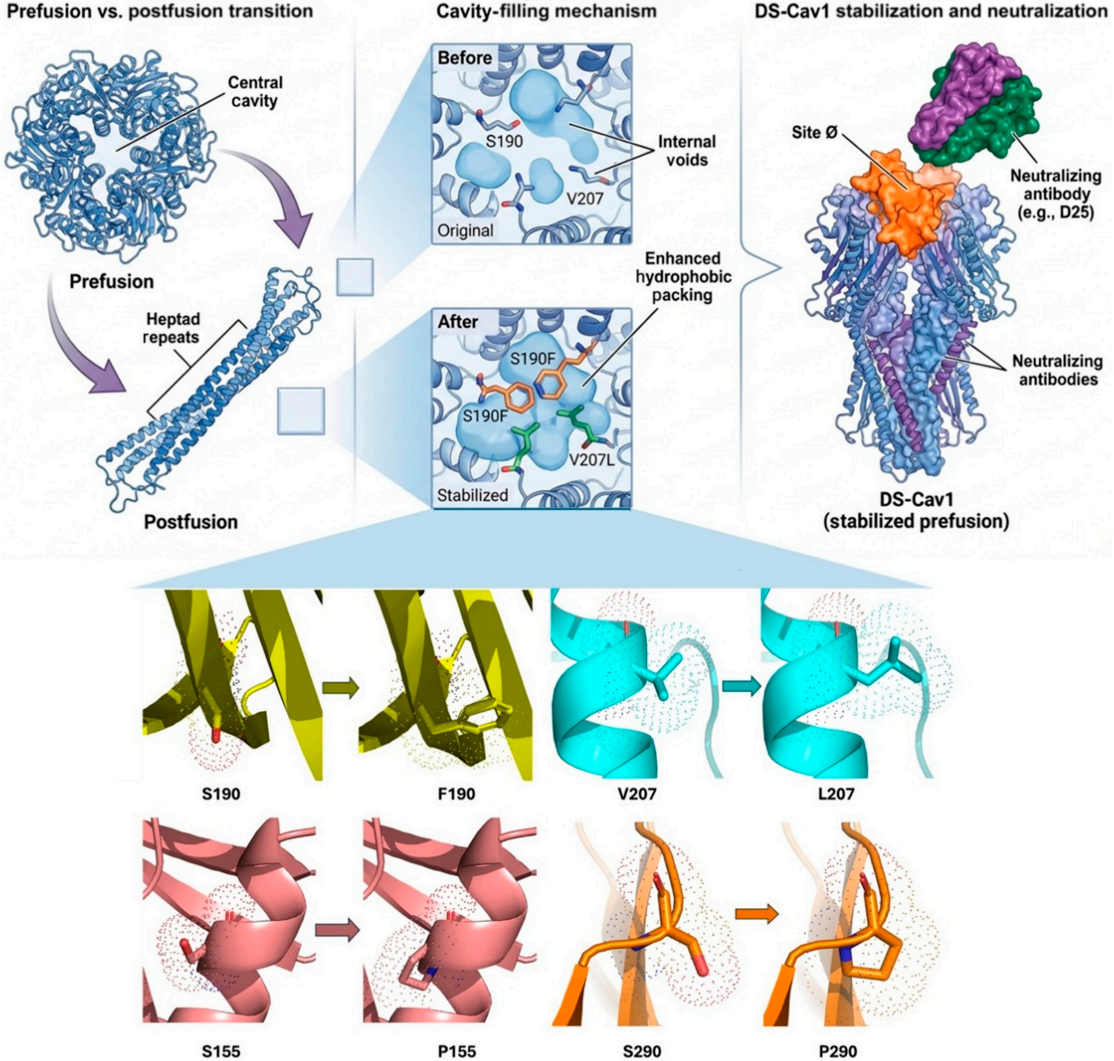
Mechanistic overview of RSV F GP stabilization via cavity-filling mutations for prefusion antigen design. The top panel shows the structural transition of the RSV F GP from a metastable prefusion state to an elongated postfusion state upon viral entry, showing the structural changes of the heptad repeat regions and the presence of a central internal void as the GP undergoes membrane fusion. Cavity-filling mutations typically involve the replacement of small or flexible residues (i.e., S190 and V207) with larger hydrophobic residues (i.e., S190F and V207L) in order to remove internal voids and increase the hydrophobic interaction between subunits, which may improve prefusion stability by increasing hydrophobic packing. The prefusion-stabilized DS-Cav1 F protein was designed to retain critical neutralization sites such as antigenic site Ø and has been reported to support binding by prefusion-specific neutralizing antibodies. The bottom panel shows residue-specific structural representations of the stabilized DS-Cav1 prefusion F protein before and after the introduction of mutations (S190F, V207L, S155P, and S290P), showing increased local packing density and decreased conformational flexibility through both steric filling and proline-mediated rigidification, thus increasing the overall prefusion stability for vaccine-oriented immunogen design.

Structural analyses have demonstrated that there are 2 areas on the prefusion trimer that contain “under-packing” regions. Those regions can potentially be stabilized by “cavity-filling mutations” (S190F and V207L). The next step was to combine those substitutions with engineered disulfide bonds (S155C-S290C) and create a new construct referred to as DS-Cav1 [4,84]. Therefore, DS-Cav1 is best considered an example of how multiple strategies for stabilizing proteins can be used in combination; specifically, it demonstrates that while the use of engineered disulfide bonds can stabilize proteins, this approach can also be complemented by using other approaches, such as cavity-filling mutations. This possible cavity-filling mutation is shown in Fig. 4.

#### DS-Cav1: The prototype design

**Fig. 5.**
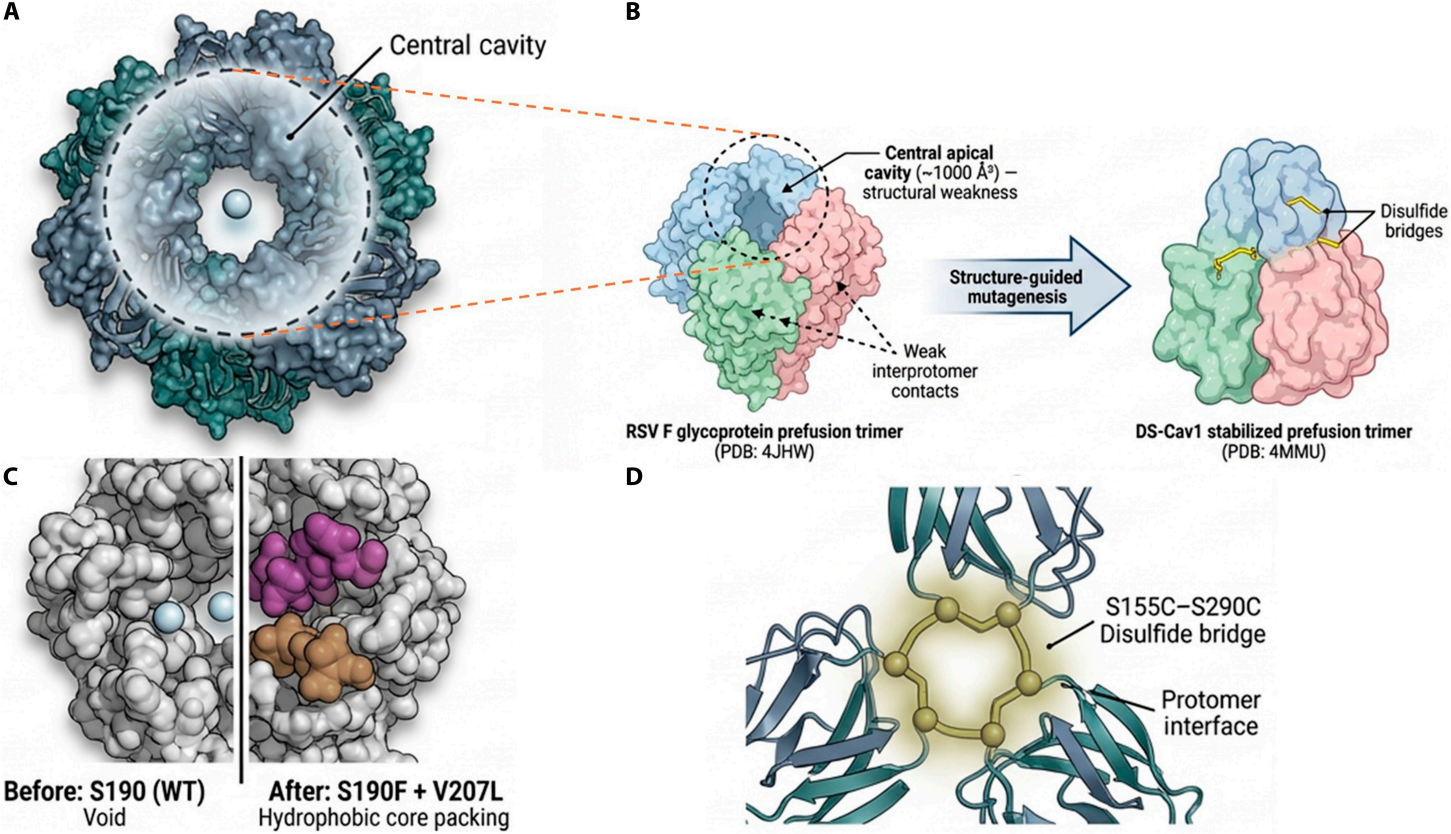
Structural basis for prefusion stabilization of DS-Cav1 RSV F GP. (A) Top view of a trimeric viral GP in a prefusion conformation with a relatively large open space or “cavity” in the middle of the 3 protomers as compared to other regions; this area is characterized by less-than-optimal packing and therefore reduced structural stability. (B) The structure of the prefusion trimer was analyzed from PDB ID: 4JHW. This analysis showed a cavity located at the apex of the trimer approximately 1,000 Å^3^ in volume along with areas where there are relatively weak interactions among the 3 protomers. (C) Comparison of internal packing pre- and post-mutation. The wild-type residue S190 creates a gap in the core, while the S190F and V207L mutations enhance hydrophobic packing, thereby occupying the cavity and stabilizing the protein core. (D) Detailed examination of the designed S155C–S290C disulfide link at the protomer interface, demonstrating its function in enhancing interprotomer contacts and stabilizing the prefusion conformation.

One of the first and most influential examples of rational prefusion stabilization is represented by DS-Cav1. The example shows how the combination of cavity-filling mutations, engineered disulfide bonds, and structural knowledge in protein engineering is used to stabilize the prefusion form. Rather than being a pure example of a cavity-filling mutation design, DS-Cav1 serves as an illustration that the strategy of filling cavities may work synergistically with other methods to maintain the prefusion form. DS-Cav1 was created for the fusion (F) protein of the RSV and has served as the foundational framework for several next-generation vaccine antigens, including those focused on SARS-CoV-2 and Nipah virus. The RSV F GP is a class I trimeric fusion protein in its prefusion state; it undergoes a large structural change from a metastable prefusion state to an elongated postfusion state upon completion of viral entry. The prefusion state was characterized in detail in the Protein Data Bank (PDB) structure 4JHW, where a central cavity approximately 1,000 Å exists at the apex of the 3-fold trimer. This hollow area, located at the apex of the trimer, serves as a point of structural weakness due to the fact that it is bordered by the remnants of each of the individual protomers that make up the trimer, allowing for premature conformational transition to the postfusion state (PDB ID: 3RRR). The detailed explanation of DS-Cav1 and the structural basis of RSV F stabilization through cavity-filling mutations are shown in Figs. 4 and 5, respectively.

Due to the presence of this internal void, in addition to weak interprotomer connections, the prefusion trimer was inherently unstable under physiological conditions and recombinantly expressed conditions. In order to reduce this instability, McLellan *et al*. [4] used structure-guided design to create specific mutations to enhance the stability of the GP. Interprotomer disulfide bridges were engineered to stabilize adjacent subunits, preventing the separation of the trimeric apex during conformational activation; S155C–S290C were chosen as the 2 interprotomer disulfide linkages.

Cavity-filling mutations were also incorporated to enhance the hydrophobic core. The greatest of these was S190F, which was positioned close to the center of the apical cavity within the F1 subunit. The replacement of the short, flexible serine side chain with the bulky aromatic phenylalanine residue greatly improved the packing density and van der Waals interactions at the interface between subunits. V207L was added to improve the local hydrophobic interactions in the same general region as S190F and further enhance the stabilizing effects of S190F. Structural comparison of the stabilized DS-Cav1 (PDB ID: 4MMU) versus the wild-type prefusion F (4JHW) demonstrated that the described design method was effective. In DS-Cav1, the phenylalanine side chain produced by the S190F substitution fills a central hydrophobic pocket that was a water-filled cavity in the wild-type structure. This mutation resulted in enhanced hydrophobic packing in the core of the prefusion shape and reduced the structural flexibility, thereby maintaining the apex in the prefusion shape. The designed disulfide linkages enhanced the interactions between protomers and resulted in a stiff and symmetrical trimer that is less likely to activate prematurely.

The recent research has provided evidence to support that optimizing the hydrophobic pocket of the RSV prefusion F trimer goes beyond the Cav1 design using the DS-Cav1 approach. The recent advancement of DS-Cav1, which combined the S190F and V207L mutations and engineered disulfide bridge stabilizing approaches, was significant in terms of prefusion stability. However, more recently, it has been demonstrated that additional hydrophobic residues can be incorporated into the prefusion trimer to provide increased structural stability, improved recombinant expression levels, and enhanced immunogenicity. It is also important to note that those variants that have had additional engineered cavity-filling mutations modifications that were designed outside of the original Cav1 design have shown greater prefusion stability when tested in vivo and elicited a significantly greater response than DS-Cav1 alone [85]. Thus, these results indicate that there are still many opportunities for refinement through continued cavity optimization by making additional hydrophobic packing improvements to improve both antigen stability and vaccine efficacy. Therefore, cavity-filling mutation engineering continues to evolve as a method to develop rationally designed vaccines.

#### Second-generation designs: DS2, SC-TM, and beyond

Following the success of DS-Cav1 (PDB ID: 4MMU) in demonstrating a method for stabilization of the prefusion form of RSV F, researchers developed second-generation RSV F constructs to improve their thermostability, manufacturability, and expression yields while preserving the native antigenic profile. The advancements made through the study of DS-Cav1, especially regarding the benefits of cavity-filling mutations and disulfide-bond formation, served as a foundation for the development of a second generation of prefusion-stabilized RSV antigens, including DS2, SC-TM, and others. The DS2 design, detailed in subsequent structure-guided studies [86,87], preserved the necessary stabilizing features of DS-Cav1, i.e., the S155C–S290C disulfide bond and the S190F/V207L cavity-filling mutations pair, but incorporated additional substitutions to improve interprotomer contacts and to remove the residual voids identified in the prefusion model. The structural characterization of DS2 revealed that although DS-Cav1 had effectively filled the major central cavity, small peripheral gaps remained in the prefusion model around the heptad repeat A (HRA) and FP regions. To address these deficiencies, the DS2 design incorporated F137W and N183F mutations into DS2. These 2 new hydrophobic substitutions were intended to fill local microcavities and to create van der Waals contacts at the top of each trimer.

**Fig. 6.**
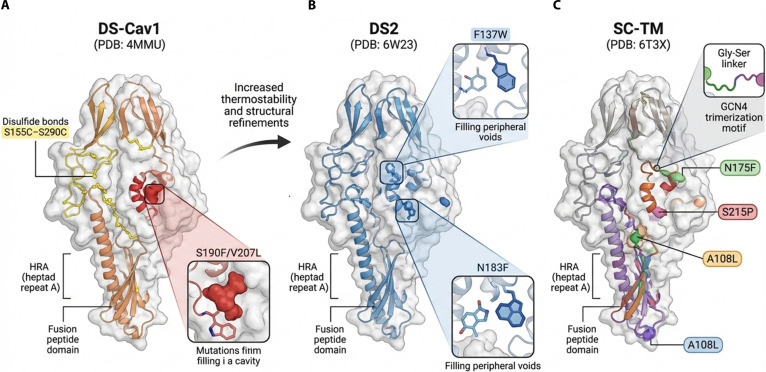
Structure-guided evolution of prefusion RSV F immunogens from DS-Cav1 to SC-TM. (A) Structure-based engineering strategies were used to progressively stabilize the prefusion conformation of the RSV fusion protein (RSV F). The first-generation construct DS-Cav1 (PDB: 4MMU) introduced a stabilizing S155C–S290C disulfide bond together with S190F and V207L cavity-filling mutations, which reduced internal voids and stabilized the prefusion structure near the heptad repeat A (HRA) and FP regions. (B) The second-generation construct DS2 (PDB: 6W23) incorporated additional hydrophobic substitutions F137W and N183F to fill residual peripheral cavities, improving internal packing, thermostability, and structural rigidity. (C) Further optimization led to the single-chain transmembrane construct SC-TM (PDB: 6T3X), which covalently links the F1 and F2 subunits via a flexible Gly–Ser linker and includes a C-terminal GCN4 trimerization motif to enhance trimer assembly. Additional stabilizing mutations (N175F, S215P, A108L) further strengthen hydrophobic packing and local rigidity, producing a highly stable prefusion antigen suitable for vaccine development.

The cryo-EM characterization of the DS2 construct (PDB ID: 6W23) demonstrated that the prefusion architecture was conserved with good fidelity [86]. The newly added F137W and N183F side chains were found to occupy small hydrophobic cavities previously occupied by ordered water molecules in DS-Cav1, thus reducing internal void volume and increasing rigidity. DS2 further enhanced prefusion stability by introducing additional interprotomer disulfide bonds that increased trimer rigidity, resulting in an approximately 8 °C increase in melting temperature (*T*_m_) compared with DS-Cav1 [88]. The evolution of RSV F protein immunogens is summarized and illustrated in Fig. 6.

To date, the most effective methods of improving stability have been developed using the single-chain TM (SC-TM) RSV F construct [86] (PDB ID: 6T3X). In addition to providing an alternate method of stabilization [86], the study provided data that showed that the 2 subunits of the F protein (F1 and F2) could be chemically cross-linked using a flexible glycine–serine (GS) linker, thereby ensuring that cleavage between them would not result in separation of the subunits. The study also showed that the construct had many of the same features as DS2, including cavity-filling mutations and proline-scanning mutations, but added a C-terminal GCN4 trimerization motif to improve trimer formation. Of particular importance in improving the structural integrity and temperature stability of this construct was the inclusion of 3 key mutation sites, N175F (cavity-filling mutations), S215P (enhanced local rigidity due to the substitution of serine to proline), and A108L (hydrophobic packing at the FP-proximal contact). These combined efforts resulted in enhanced structural uniformity and thermal stability, and cryo-EM studies showed that there was increased density at the apex region, indicative of reduced flexibility. Building on these previous studies and approaches, several next-generation RSV F constructs utilize a systematic proline-scanning approach across all hinge regions and flexible loop domains. Similar to the previously described proline-scanning approach for SARS-CoV-2 S-2P and HexaPro S proteins, these studies found sets of proline substitutions numbering from 5 to 7 that provide similar or superior stabilization compared to traditional cavity-filling mutation designs. Additionally, many of the proline-modified variants expressed higher levels than the corresponding wild-type sequences and showed enhanced thermal stability, while others required further optimization to maintain intact epitopes at antigenic site Ø. Collectively, the RSV prefusion F antigens (DS2, SC-TM, and 7P designs) represent the culmination of approximately 10 years of continued refinement of prefusion F protein-based vaccines. Each successive generation has utilized high-resolution structural information primarily obtained from the PDB entries 4MMU, 6W23, and 6T3X to overcome residual liabilities present in the prefusion conformation. The resulting constructs have exhibited remarkable thermostability, uniform prefusion homogeneity, and induced potent neutralizing antibodies in multiple animal models.

These advances have greatly facilitated the successful development and licensure of the RSV vaccines currently used in maternal vaccination programs and older adults, such as Pfizer’s Abrysvo and GSK’s Arexvy, each of which contains prefusion-stabilized F antigens derived from DS-Cav1 and its derivatives. The progression from DS-Cav1 to DS2 and subsequent variants illustrates how cavity-filling mutation engineering, when combined with complementary stabilization strategies, can improve prefusion stability in RSV F proteins.

### SARS-CoV-2 S protein: Cavity-filling mutations within a multi-strategy stabilization framework

**Fig. 7.**
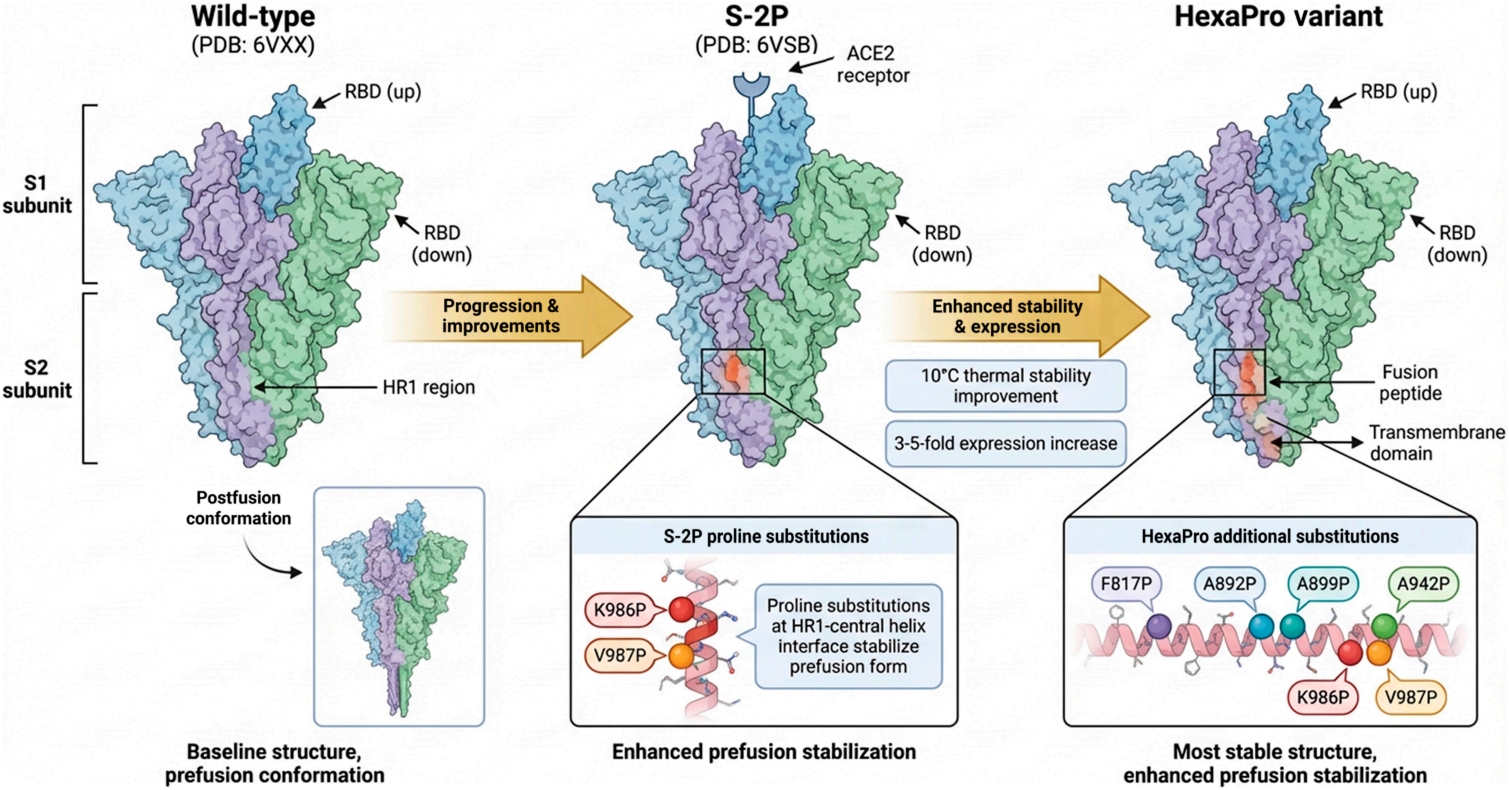
Structure-guided stabilization of the SARS-CoV-2 S protein. The wild-type S GP (PDB: 6VXX) is composed of S1 and S2 subunits and adopts receptor-binding domain conformations in the “up” and “down” states. In order to maintain a prefusion structure in the S-2P variant (PDB: 6VSB), 2 mutations (K986P and V987P) were made in the central helix region of HR1 to prevent uncontrolled folding into its final configuration while allowing it to bind to ACE2. Additional prolines (F817P, A892P, A899P, and A942P) were introduced to create the HexaPro variant. The hexaproline mutations have enhanced the structural integrity of the S, increased the thermal resistance of the S by approximately 10 °C, and increased the protein yield by 3 to 5 times as compared to previous variants. This has enabled vaccine developers to utilize this protein for use in vaccines and also provides stable structural samples for structural studies.

The SARS-CoV-2 S GP is a trimeric class I fusion protein that facilitates viral entry by binding to the host cell’s ACE2 receptor. As a class I fusion protein, the S is metastable in its prefusion form and undergoes conformational rearrangement to a postfusion form. To generate a structurally intact, immunogenic antigen for COVID-19 vaccines, it was necessary to stabilize the prefusion form of the S. Although the key SARS-CoV-2 S stabilizing mutations are summarized in Table 4, the major 2P and HexaPro have been summarized in more detail. In addition, the SARS-CoV-2 S protein stabilization evolution is illustrated in Fig. 7.

**Table 4.** Key SARS-CoV-2 S stabilizing mutations

Design	Mutation(s)	Mechanism	Representative PDB ID	Stability improvement
Wild-type S		Metastable, transitions to postfusion	6VXX	
S-2P	K986P, V987P	Hinge rigidification, partial cavity reduction	6VSB	+10 °C *T*_m_, ↑ yield
HexaPro (S-6P)	F817P, A892P, A899P, A942P, K986P, V987P	Multi-hinge rigidification, cavity-filling mutations	6XKL	+15 °C *T*_m_, 10× yield
VFLIP	K986P/V987P + hydrophobic substitutions (e.g., Q1005L)	Cavity-filling mutations, improved interprotomer packing	7BNN	↑ thermostability
S2GΔHR2	Proline + aspartate substitutions (e.g., A942D)	Prefusion bias via postfusion destabilization	7BNO	↑ stability, ↓ flexibility
HR1–CH interface stabilized variant	L938F/L938Y	HR1–CH interface	6XKL	HexaPro core packing region
Central helix variant	T941I/T941V	Central helix	6XKL	S2 hinge stabilization
S2 core bundle variant	V963L/V963F	S2 3-helix bundle	7BNN	VFLIP internal packing
FPPR hydrophobic variant	I870F	FPPR region	7BNO	S2GΔHR2 fusion-proximal zone
HR1 micro-cavity variant	A893F/A893L	HR1	6XKL	Local cavity sealing
Aromatic packing variant	G885F	HR1 loop	6XKL	Helical bundle contact
Fusion-adjacent variant	T912I	HR1–FP interface	7BNN	Interhelical packing
SD2–S2 junction variant	V705L	SD2–S2 boundary	6VSB	Prefusion hinge contact
Trimer core variant	A1020L	Central trimer interface	7BNO	Interprotomer stability
S1–S2 interface variant	T547V	Protomer contact region	6VSB	S1–S2 cavity site

#### S-2P: Rapid translation to clinical success

The S-2P antigen design involves 2 sequential proline substitutions (K986P and V987P) located in the hinge region 1 (HR1)–central helix (CH) interface of the S ectodomain [24]. These residues are positioned near the tip of the S2 subunit, where there is significant conformational movement during fusion. Proline substitutions were chosen for their ability to restrict backbone flexibility and stabilize the S2 subunit local bend while maintaining its structure in HR1 compared to CH. Proline also prevents the premature transition into the postfusion state, as seen in previous fusions. It is important to note that both structural and computational studies have shown that the K986P and V987P mutations increase the stability of this area by increasing hydrophobic contact between the core residue and the adjacent residues, in addition to decreasing the cavity size in this same area due to a decrease in potential areas of cavitation surrounding residues 985 to 988.

The S-2P prefusion stabilized ectodomain (denoted as PDB ID: 6VSB) adopts a well-ordered trimeric configuration with receptor-binding domains (RBDs) in both “up” and “down” conformations. Compared with wild-type S (PDB ID: 6VXX), S-2P exhibited approximately a 10 °C increase in thermal stability and a 3- to 5-fold improvement in recombinant yield in mammalian expression systems under the experimental conditions evaluated [24]. Subsequent optimization led to the HexaPro variant, which further improved protein stability and expression while maintaining the prefusion conformation. S-2P was the antigenic basis for multiple approved COVID-19 vaccines: mRNA-1273 (Moderna), BNT162b2 (Pfizer/BioNTech), and NVX-CoV2373 (Novavax, protein subunit vaccine). Cryo-EM structures of S-2P complexes with neutralizing antibodies (e.g., PDB ID: 7BNM) demonstrated that the stabilized trimer accurately presented critical epitopes on the virus surface in the prefusion conformation, including the receptor-binding site, the N-terminal domain (NTD) supersite, and the S2 apex. S-2P exemplifies one of the fastest and most successful transitions of structure-based vaccine development to the clinic.

#### Next-generation stabilization: HexaPro and beyond

The antigen design for S-2P has included 2 consecutive proline substitutions (K986P and V987P) at the HR1–CH interface of the S ectodomain S2 region [24]. The proline substitutions were located at the tip of the S2 subunit, which is an area of significant conformational flexibility upon fusion of the virus into host cells. The proline substitution restricts the conformational movement of the peptide backbone and maintains the local bend, distinguishing the HR1 from the CH, and prevents the premature transition to the postfusion state. Of importance, both structural and computational studies demonstrated that the proline substitutions also had an indirect effect to stabilize the surrounding cavities within the central core through enhanced hydrophobic interactions between residues in the core and prevented the creation of cavities around the residues 985 to 988. The prefusion-stabilized S-2P ectodomain (denoted as PDB ID: 6VSB) contained a highly ordered trimeric configuration with all 3 “up” or “down” configurations of the RBDs. In comparison to wild-type S (PDB ID: 6VXX), S-2P displayed an increase of approximately 10 °C in thermal stability and a 3- to 5-fold increase in recombinant yield when produced using mammalian cell-based expression systems. S-2P served as the antigenic basis for the first set of approved COVID-19 vaccines: mRNA-1273 (Moderna), BNT162b2 (Pfizer/BioNTech), and NVX-CoV2373 (Novavax, protein-subunit vaccine). Cryo-EM structures of S-2P complexes with neutralizing monoclonal antibodies (e.g., PDB ID: 7BNM) confirmed that the stabilized trimer accurately presented the critical epitopes of the virus surface in the prefusion conformation, including the receptor-binding site, the NTD supersite, and the S2 apex. S-2P represents one of the fastest and most successful examples of structure-based vaccine development to the clinic. Beyond the widely used 2P and HexaPro S stabilization strategies, next-generation SARS-CoV-2 S designs such as VFLIP have further improved prefusion stability [89]. These modifications improved both the thermal stability and structural integrity of the antigens while preserving the structural epitopes necessary for neutralization.

### Broader applications of cavity-filling stabilization across viral vaccine development

The application of cavity-filling stabilization in viral vaccine antigen engineering is not limited to RSV F and SARS-CoV-2 S proteins. Hydrophobic core optimization strategies that are guided by the 3-dimensional structure of a viral envelope GP have also been employed in HIV-1 Env trimer structures to improve the stability of the prefusion form and to protect or maintain structural epitopes that induce neutralization. Structural analysis of influenza HA has shown similar improvements in stability through hydrophobic packing optimizations designed to reduce the conformational flexibility required for fusion activation. Metapneumovirus F protein engineering has used structural similarity to RSV F to implement optimization of the same types of structural features that were optimized in RSV F. Optimization approaches similar to those described above have provided additional contributions to stabilizing efforts involving Nipah virus fusion GPs, parainfluenza virus fusion proteins, filoviral GPs, and others. Improving hydrophobic packing and minimizing internal structural instability may contribute to the enhancement of recombinant expression yields and improve the overall antigen stability. Importantly, cavity-filling mutations often include one or more other stabilization methodologies such as proline substitution, engineered disulfide bridges, interprotomer locking mutations, and/or helix-stabilizing mutations. Therefore, cavity-filling mutation engineering may be considered a structural vaccinology strategy that has demonstrated utility across multiple viral systems.

### Challenges and current limitations of cavity-filling stabilization approaches

Although there has been significant success with the use of cavity-filling mutations to provide a stable environment for metastable prefusion viral GPs, the effectiveness of this strategy is limited by several factors. The degree to which viral fusion proteins can be stabilized using cavity-filling mutations varies; certain types of viral fusion proteins are less responsive to cavity-filling mutations due to instability arising from electrostatic frustration, backbone strain, or large-scale domain movement rather than internal packing defects. Additionally, over-stabilization may result in reduced conformational flexibility and thus reduced ability to present antigens optimally or engage receptors appropriately and induce protective immune responses. Over-stabilization could also affect access to epitopes or distort the native antigenic structure. Larger hydrophobic amino acids used to fill cavities can sometimes cause steric hindrance or interfere with correct folding of the protein, and can lower recombinant expression levels. Finally, the accurate identification of cavities depends upon having structural resolution data and computational predictive models of sufficient accuracy, which limits applicability to unstructured viral target structures. Therefore, successful implementation will likely require iterative optimization of combinations of computational modeling, structural validation, and immunological evaluation to determine a suitable balance between antigenic structure stability and fidelity.

Several methods have been developed to engineer metastable prefusion viral fusion proteins that have different mechanisms to address structural determinants of conformational instability. For example, cavity-filling mutations can help create a more stable hydrophobic core by reducing internal empty space. Proline substitution can limit backbone flexibility, and disulfide-bond formation can limit large-scale conformational changes. Recently, AI-assisted protein design and combinatorial engineering strategies have emerged as ways to identify synergistic sets of stabilizing mutations that provide additional improvements in terms of antigen stability, expression, and manufacturability. While direct quantitative comparison among different viral systems is difficult due to variations in protein architectures and experimental conditions, overall trends emerge from studies that compare different stabilization approaches. A summary of many stabilization approaches compared is shown in Table 5.

**Table 5.** Comparative analysis of cavity-filling mutations, proline-based, disulfide-bond, and emerging stabilization strategies for viral fusion proteins

Strategy	Mechanism	Typical stability gain	Advantages	Limitations	Examples
Cavity-filling mutations	Improves hydrophobic packing	Moderate–high (often 5–20 °C *T*_m_ increase)	Often preserves antigenicity in reported systems	Requires structural information	RSV DS-Cav1, SARS-CoV-2 S
Proline substitutions	Restricts backbone flexibility	Moderate–high	Simple and widely applicable	May affect expression/folding	2P, HexaPro
Disulfide engineering	Locks domains in place	Moderate–high	Strong conformational restraint	Possible mispairing	DS-Cav1, SARS-CoV-2 S2D
AI/computational design	Multi-parameter optimization	Variable, often synergistic	Explores broader sequence space	Requires computational validation	ProteinMPNN, RFdiffusion-guided designs

Cavity-filling mutations have been successful in many viral fusion protein systems; however, they are not universally effective. Over-stabilization of the GP could reduce its conformational flexibility needed for optimal antigen presentation, receptor binding, or generation of protective immune responses. Stabilizing mutations can also alter the accessibility of epitopes or make subtle alterations to the native antigenic architecture, and thus negatively impact immunogenicity. Predictive algorithms currently available for identifying effective cavity-filling mutations are challenged by 2 primary issues. First, the stabilizing effects generated by these mutations are generally dependent on specific contexts that include multiple interacting elements within the structure. Second, it can be difficult to predictively model epistemically interacting elements based on computational predictions alone. Additionally, the presence of large amounts of glycoconjugates or glycans on highly glycosylated viral GPs, including HIV-1 Env, Lassa virus GP, and certain coronavirus S variants, creates numerous obstacles for predicting effective cavity-filling mutations. These obstacles include obscuring structurally relevant areas of the protein surface with glycans, influencing local conformational dynamics caused by glycans positioned close to candidate cavity sites, limiting access to candidate cavity sites by shielding them from solvent exposure. To overcome these challenges in the future and expand the scope of application of cavity-filling mutation-based stabilization strategies in next-generation vaccine development, integration of glycan-sensitive structural modeling tools with AI-guided protein design tools, deep mutational scanning techniques, and experimental verification tools will be critical.

## Future Perspectives

The use of AI is anticipated to further enhance the speed at which new vaccines are developed based upon their 3-dimensional structures, with AI potentially accelerating this process by better predicting the mutations that will most effectively stabilize the proteins, providing optimal configurations for the packing of residues within proteins, and identifying potential candidates for experimental verification. The 3 primary strategies for stabilizing prefusion proteins include using mutations that fill cavities, substituting residues with prolines, and creating engineered disulfide bridges. These different types of mutations produce structural stabilization via separate and complementary pathways. As such, researchers have begun combining these approaches to create stabilized forms of prefusion proteins beyond what could be achieved by individually implementing each approach.

In order to continue advancing cavity-filling protein engineering, it is anticipated that future research will utilize iterative computer-experimental workflows as opposed to singular prediction techniques. Researchers anticipate utilizing generative protein design tools such as ProteinMPNN and RFdiffusion to generate lists of proposed cavity-filling mutations, AlphaFold3 to evaluate the structural compatibility of these mutations, MD simulations and physics-based energy calculation tools (such as Rosetta, FoldX, and free-energy perturbation) to determine the degree of stabilization provided by the proposed mutations, and deep mutational scanning to provide experimental data used to refine subsequent generations of optimized cavity-filling mutations.

While significant advancements have been made in the area of cavity-filling protein engineering, there are still several challenges facing researchers in this field. Some of the specific challenges that face researchers working in this field include generating mutations that overly stabilize the protein and reduce its ability to undergo conformations necessary for function or binding with an antibody, generating mutations that alter the epitope recognized by neutralizing antibodies, and developing effective cavity-filling mutations algorithms that account for the extensive glycan shielding present on viral GPs such as HIV-1 Envelope (Env) and Lassa virus GP. It is anticipated that continued advancements in both the use of AI-assisted protein engineering and integrated structural modeling will significantly contribute to overcoming some of these challenges.

## Ethical Approval

No animal or human is involved in this study, and the manuscript does not require ethical approval.

## Data Availability

No data have been associated with this article.
